# Cardiovascular risk factors and Parkinson's disease in 500,000 Chinese adults

**DOI:** 10.1002/acn3.732

**Published:** 2019-03-10

**Authors:** Jennifer Kizza, Sarah Lewington, Benjamin Mappin‐Kasirer, Iain Turnbull, Yu Guo, Zheng Bian, Yiping Chen, Ling Yang, Zhengming Chen, Robert Clarke, on behalf of the China Kadoorie Biobank

**Affiliations:** ^1^ Clinical Trial Service Unit and Epidemiological Studies Unit (CTSU) Nuffield Department of Population Health University of Oxford Oxford United Kingdom; ^2^ Chinese Academy of Medical Sciences Beijing China

## Abstract

**Objective:**

The objectives of this study were to compare the risks of Parkinson's disease among those with versus those without prior stroke or heart disease at baseline in a prospective study of 0.5 million adults in China, and to examine associations of cardiovascular disease risk factors (cigarette smoking, hypertension, diabetes, obesity) with risk of Parkinson's disease.

**Methods:**

During an average of 11.5 years of follow‐up of 503,497 middle‐aged participants in the China Kadoorie Biobank study, 603 incident cases were hospitalized with a diagnosis of Parkinson's disease. Cox proportional hazards models were used to assess associations of history of heart disease or stroke with Parkinson's disease in all participants, and of cardiovascular disease risk factors with Parkinson's disease in a subset without prior cardiovascular disease.

**Results:**

In this population the incidence rate of Parkinson's disease (mean [SD] age of cases, 61 [10] years) was 13.3 (95% confidence interval: 12.3–14.4) per 100,000 person‐years. Incidence increased with age, and was higher in men than in women, and in urban than in rural residents. Prior stroke was associated with about twofold higher risk of Parkinson's disease (hazard ratio 1.94; 1.39–2.69). After adjustment for confounders in those without prior cardiovascular disease, a 5 kg/m^2^ higher body mass index was associated with 17% (1.17; 1.03–1.34: *P *=* *0.019) higher risk of Parkinson's disease, but neither hypertension, diabetes, nor current cigarette smoking was significantly associated with Parkinson's disease.

**Interpretation:**

Prior stroke and adiposity were each associated with higher risks of Parkinson's disease, but none of the other cardiovascular disease risk factors were significantly associated with Parkinson's disease in this population.

## Introduction

Parkinson's disease (PD) affects about 1–2% of the adult population aged 60 years or older in China,^1^, ^2^, ^3^ suggesting that about 1.7–2.0 million older people are likely to suffer from PD. Despite the fact that China has the largest population of older people and the largest number of PD cases in a single country, little is known about the determinants of PD in the Chinese population.^2^ Previous studies have reported different incidence rates of PD in Asian compared with Western populations, but few studies have assessed the determinants of PD in Asian populations.^2^, ^4^, ^5^, ^6^, ^7^, ^8^, ^9^ Prospective studies, conducted in Western populations, have reported discrepant results for the associations of cardiovascular disease (CVD) risk factors with PD, with inverse associations with smoking, but positive associations with other CVD risk factors.^2^, ^10^, ^11^, ^12^, ^13^, ^14^, ^15^, ^16^, ^17^, ^18^, ^19^, ^20^, ^21^, ^22^, ^23^, ^24^, ^25^, ^26^, ^27^, ^28^, ^29^, ^30^ Hence, further studies of risk factors for PD in Asian populations should be informative about the effects of modifiable risk factors for PD worldwide.

The aims of this study were to: (1) compare the risks of PD among those with vs. those without prior stroke or heart disease at baseline in the China Kadoorie Biobank (CKB) study; and (2) examine the associations of cigarette smoking, obesity, hypertension, and diabetes with risk of PD in a subset without prior history of CVD at baseline before and after adjustment for relevant confounding factors.

## Methods

### Standard protocol approvals, registrations, and patient consents

Ethics approval was obtained from the relevant international, national, and local ethics committees.^31^

### Participants and data collection

The CKB study is a prospective cohort study of 512,891 men and women, aged 30 to 79 years, who were recruited from five urban and five rural centers in China between 15 July 2008 and 25 June 2014. At enrollment, data were collected on demographic, medical history, and lifestyle characteristics using an interviewer‐administered electronic questionnaire.^31^

Demographic and behavioral characteristics included details on age, sex, region, income, occupation, education status, alcohol consumption, cigarette smoking, and physical activity.^32^ Self‐reported medical history included responses to questions asking if they “had ever been diagnosed by a doctor as having” either diabetes, hypertension, ischemic heart disease (IHD), stroke (including transient ischemic attack: TIA), cancer, or head injury, respectively. Clinical examination included measurements of weight (kg), standing height (mm), waist and hip circumference (mm), blood pressure (mmHg), and all participants had blood collected for nonfasting plasma glucose (mmol/L) concentrations.^8^, ^32^, ^33^

A physician coded all identified free‐text descriptions of PD in health insurance records for each hospitalization using the International Classification of Diseases 10th Revision (ICD‐10) code of G20 for idiopathic PD. Descriptions that indicated any secondary causes of Parkinsonism (G21) or Parkinsonism in diseases classified elsewhere (G22) or other diseases of the basal ganglia or other extrapyramidal disorders (G23‐G26) were excluded from analyses in this study.

### Statistical analyses

Participants with missing data or biologically implausible outliers were excluded, as were those with a prior history of head injury (to exclude secondary causes of PD) or cancer (with the exception of nonmelanoma skin cancer) at baseline. Height, weight, body mass index (BMI; kg/m^2^), and waist‐hip ratio were categorized into quintiles to assess the shape of any associations with PD. Individuals were categorized by the presence or absence of hypertension at baseline (systolic blood pressure [SBP] ≥140 mmHg, diastolic blood pressure [DBP] ≥90 mmHg, or receiving treatment for hypertension).^8^ Presence of diabetes at baseline was defined as reported medical history of diabetes with a fasting blood glucose level ≥7.0 mmol/L, or a random blood glucose measurement ≥11.1 mmol/L and a fasting time <8 h, or a random blood glucose measurement ≥7.0 mmol/L and a fasting time >8 h.^33^ Individuals were categorized by smoking status (never smoker, occasional smoker, ex‐regular smoker who quit due to illness, other ex‐regular smoker, or current smoker including ex‐regular smokers who quit smoking in the last 5 years)^4^ and amount of cigarettes smoked per day (0, <20, 20+ cigarettes per day). Individuals were also categorized by presence or absence of obesity (body mass index [BMI] ≥30 kg/m^2^).^34^

Incidence rates of PD were calculated by age at baseline, sex, and region. Cox proportional hazards models were used to assess the associations of prior CVD (ischemic heart disease [IHD], or stroke [including TIA]) with PD in all participants. To minimize potential reverse causality bias, all analyses of CVD risk factors (smoking, adiposity, hypertension, and diabetes) were conducted in a subset of participants with no prior history of IHD or stroke at baseline.^33^ All models were adjusted for current age (i.e., time‐updated age), sex, and region.^35^, ^36^ Associations of PD with main exposures (IHD, stroke/TIA, smoking, cigarettes per day, height, weight, BMI, waist‐hip ratio, obesity, hypertension, and diabetes) were assessed after adjustment for covariates (current age, sex, and region), and for other confounders (income, education, occupation, alcohol consumption, physical activity as Metabolic Equivalent of Task [MET]^32^).

The hazard ratios (HR) were estimated for a one standard deviation (SD) difference for continuous exposures. All models were tested for effect modification by current age, sex, and region.^9^, ^27^, ^35^ For all categorical variables with more than two levels, risk estimates were accompanied by group‐specific 95% confidence interval (CI), representing the statistical information derived only from such groups.^37^ Population attributable fractions were estimated to quantify the importance of each risk factor for PD.^38^

Sensitivity analyses for associations of adiposity measures with PD were conducted both in a subset of never smokers, and those with or without obesity (BMI ≥25 kg/m^2^).^34^, ^39^ Analyses were also repeated after excluding the first 3 years of follow‐up to minimize effects of reverse causality. Analyses were conducted using STATA 14 software (StataCorp, College Station, TX, USA) and figures were plotted using R 3.4.0 software (R Foundation for Statistical Computing, Vienna, Austria).

## Results

### Characteristics of the study population

After exclusion of individuals with missing data or outliers, 503,497 (98.2%) participants were available for the present analyses. Among these participants, 603 had an incident PD event. Analyses of the associations of CVD risk factors with PD were restricted to the 480,950 participants with no prior history of CVD and included 521 PD cases (Table S1). The mean duration of follow‐up was 9 years in all participants.

Overall, 59.2% of participants were women, 56.0% lived in rural regions, 57.3% had an income less than or equal to 19,999 yuan, and 50.8% had a primary school education (up to age 11 years) or lower. Mean age of PD cases was older than that of the general population (60.9 vs. 51.5 years), and cases had a lower level of education (62.2% vs. 50.8%). Likewise, a higher proportion of PD cases were agricultural workers or were retired compared with the general population (68.5% vs. 58.3%).

Most participants were never regular drinkers (46.0%) or occasional drinkers (31.9%). However, PD cases reported lower mean (SD) levels of physical activity than all participants (14.8 [11.8] vs. 21.1 [13.9] MET‐hours/day). While 61.2% of men were current smokers, only 5% of women smoked, hence, analyses of smoking were restricted to men. At baseline 5.9% of all participants had diabetes, 32% were overweight or obese (BMI ≥25 kg/m^2^), and 33.5% had hypertension.

### Incidence of hospitalized cases of Parkinson's disease

The incidence rate of PD was 13.3 (95% CI: 12.3–14.4) per 100,000 person‐years in all hospitalized participants (Table 1), but incidence rate varied by age, sex, and region. The incidence rate increased with age from 1.6 (1.1–2.4) per 100,000 person‐years in those aged ≤50 years at baseline, to 48.8 (43.2–55.0) per 100,000 person‐years for participants over 70 years of age at baseline. PD incidence rates were higher in men (15.6; 13.9–17.5 per 100,000 person‐years) than in women (11.7; 10.5–13.1 per 100,000 person‐years). Similarly, PD incidence rates were slightly higher in urban (14.8; 13.2–16.6 per 100,000 person‐years) than in rural regions (12.1; 10.9–13.6 per 100,000 person‐years).

**Table 1:** Parkinson's disease incidence rates, by age, sex, region, and overall

Variable	Events	Person‐years	Rate (95% CI) per 100,000 person‐years
Age			
≤50	25	1,549,464	1.6 (1.1–2.4)
>50 – ≤60	106	1,426,211	7.4 (6.1–9.0)
>60 – ≤70	206	1,019,392	20.2 (17.6–23.2)
>70	266	545,398	48.8 (43.2–55.0)
Sex			
Men	284	1,821,363	15.6 (13.9–17.5)
Women	319	2,719,103	11.7 (10.5–13.1)
Region			
Urban	292	1,977,448	14.8 (13.2–16.6)
Rural	311	2,563,018	12.1 (10.9–13.6)
Total	603	4,540,466	13.3 (12.3–14.4)

### Associations with cardiovascular disease

Prior history of IHD was associated with a 44% higher risk of PD after adjustment for current age, sex, and region when compared with participants without prior medical history of IHD at baseline (HR 1.44; 95% CI: 1.07–1.93). However, there was no association of IHD with risk of PD after additional adjustment for relevant confounders (Table 2).

**Table 2:** Associations of ischemic heart disease and stroke with Parkinson's disease

	Events/ participants	HR (95% CI)		
		Adjusted for age‐at‐risk, sex, region	+Income, occupation, education	Fully adjusted^1^
Ischemic heart disease				
No	553/488,415	1.00 (‐)	1.00 (‐)	1.00 (‐)
Yes	50/15,082	1.44 (1.07–1.93)	1.26 (0.93–1.69)	1.20 (0.89–1.61)
*Heterogeneity* 2 *(χ* ^2^ *, p)*	‐	5.34, 0.021	2.15, 0.143	1.31, 0.253
Stroke^3^				
No	563/494,847	1.00 (‐)	1.00 (‐)	1.00 (‐)
Yes	40/8,650	2.22 (1.61–3.07)	2.05 (1.48–2.84)	1.94 (1.39–2.69)
*Heterogeneity* 2 *(χ* ^2^ *, p)*	‐	18.78, <0.001	15.34, <0.001	13.00, <0.001

1 HR further adjusted for baseline smoking, alcohol consumption, physical activity, BMI, SBP, DBP, and diabetes.

2 Test for heterogeneity of HR by categories of the main exposure, conducted using likelihood ratio tests with 1 degree of freedom.

3 Stroke defined as participants with reported medical history of stroke or TIA at baseline.

In contrast, prior stroke was associated with a twofold higher risk of PD after adjustment for current age, sex, and region, when compared with participants without prior history of stroke at baseline (HR 2.22; 95% CI: 1.61–3.07), and was only slightly attenuated after additional adjustment for all confounders (1.94: 1.39–2.69) (Table 2). Assuming a history of stroke is causal for PD, the population attributable fractions based on the fully adjusted HR for stroke/TIA suggested that a history of stroke accounted for approximately 9.4% of all hospitalized cases of PD.

### Associations with smoking (men only)

**Figure 1:**
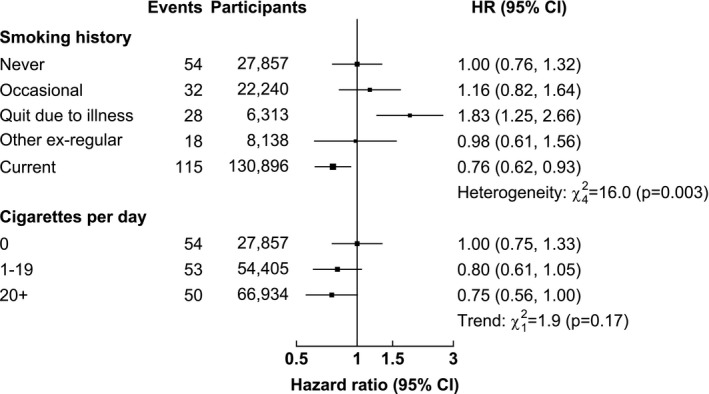
Associations of smoking with incidence of Parkinson's disease. Analyses conducted in men only. Models adjusted for age‐at‐risk, region, education, occupation, income, alcohol consumption, physical activity, BMI, SBP, DBP, and diabetes. HR are reported with group‐specific 95% CIs. For updated smoking, current smokers were defined as regular smokers or ex‐regular smokers who quit smoking ≤5 years ago at baseline. Cigarettes per day analyses excluded ex‐smokers and occasional smokers.

After adjusting for current age and region, current smoking was inversely associated with risk of PD (*χ* ^2 ^= 24.38, *P* < 0.001), but this association was attenuated, albeit remained significant, after adjustment for relevant confounders (*χ* ^2 ^= 16.04, *P* = 0.003; Fig. 1, Table S2). Compared with never smokers (HR 1.00; group‐specific 95% CI: 0.76–1.30), current smokers had 39% lower risk of PD (0.61: 0.50–0.74, p_het_ = 0.004) after adjustment for age, sex, and region, but this was attenuated to a 24% lower risk and was no longer statistically significant after additional adjustment for other confounders (0.76: 0.62–0.93, p_het_ = 0.116). There was a nonsignificant trend for a more extreme inverse association with higher amounts of daily cigarette consumption (*χ* ^2^ = 1.90; *P *=* *0.168; Fig. 1). After adjustment for other confounders, men who had quit smoking due to illness were at higher risk (1.83: 1.25–2.66) than either never smokers (1.00: 0.76–1.32) or current smokers (0.76: 0.62–0.93; Fig. 1). The associations were slightly attenuated after exclusion of the first 3 years of follow‐up (Table S3).

### Associations with anthropometric measurements

**Figure 2:**
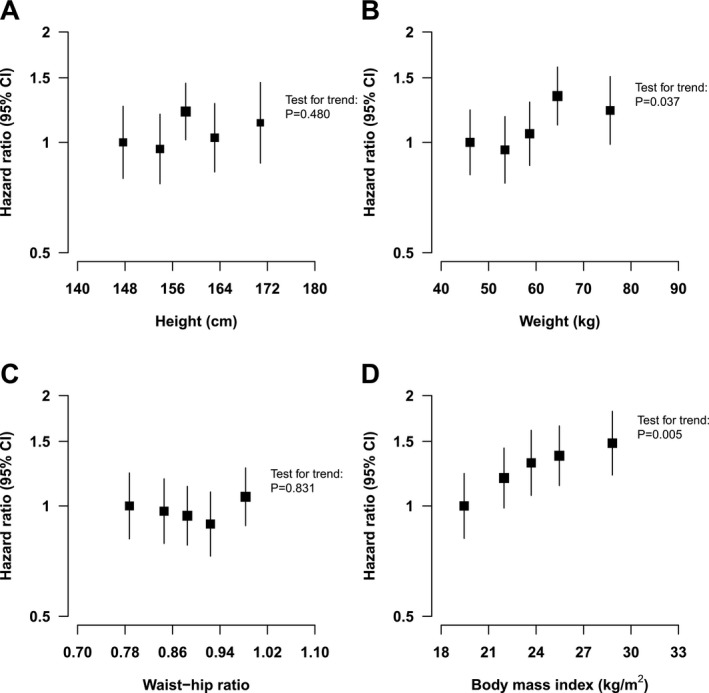
Associations of anthropometric measures with incidence of Parkinson's disease. HR for all models adjusted for age‐at‐risk, sex, region, education, occupation, income, baseline smoking, alcohol consumption, physical activity, SBP, DBP, and diabetes. HR are reported with group‐specific 95% CIs. (A) Associations of quintiles of height with incidence of PD. (B) Associations of quintiles of weight with incidence of PD. HR (95% CI) per each 15 kg higher weight is 1.16 (1.01–1.33). (C) Associations of quintiles of waist‐hip ratio with incidence of PD. (D) Associations of quintiles of BMI with incidence of PD. HR (95% CI) per each 5 kg/m^2^ higher BMI is 1.17 (1.03–1.34).

There was no association of waist‐hip ratio or height on PD risk (*P* all > 0.05; Table S4). There was a positive association of weight on PD risk (*P* = 0.006), which was attenuated after adjustment for other confounders, each 15 kg higher body weight was associated with 16% higher risk (HR 1.16: 95% CI: 1.01–1.33, *P *=* *0.036; Fig. 2).

Likewise, after adjustment for age, sex, and region, there was evidence for a significant, positive effect of BMI on PD risk (*P *=* *0.002), which was attenuated after further adjustment for other confounders: each 5 kg/m^2^ higher BMI (approximately equivalent by 1 SD change to a 15 kg increase in weight) was associated with 17% higher risk (HR 1.17: 95% CI: 1.03–1.34, *P *=* *0.019).

Sensitivity analyses conducted in the subset of never smokers indicated that there was evidence of an effect of BMI on PD risk after adjustment for confounders (*P* = 0.007). Likewise, after adjustment for confounders, each 5 kg/m^2^ higher BMI was associated with 17% higher risk (HR 1.17: 95% CI: 1.01–1.39, *P *=* *0.048). However, in a sensitivity analysis of associations of weight and PD in never smokers, a test for trend of HR following adjustment for confounders was not statistically significant (*P *>* *0.05). The associations between anthropometric measures and PD risk were slightly attenuated following exclusion of the first 3 years of follow‐up (Table S4).

### Associations with different CVD risk factors

**Figure 3:**
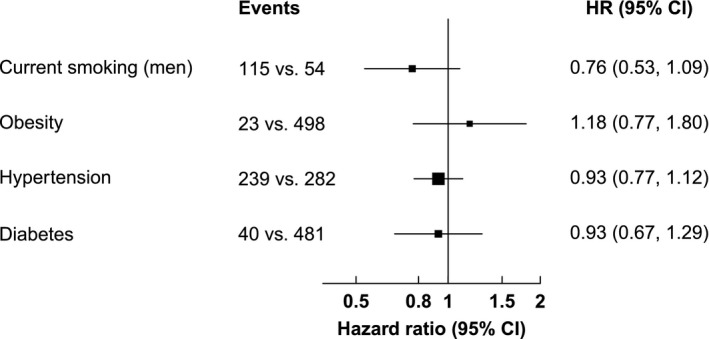
Associations of smoking, obesity, hypertension, and diabetes with incidence of Parkinson's disease. HR for all models adjusted for age‐at‐risk, region, income, education, occupation, alcohol consumption, and physical activity. HR for baseline and updated smoking additionally adjusted for BMI, SBP, DBP, and diabetes. HR for obesity further adjusted for sex, baseline smoking, SBP, DBP, and diabetes. HR for hypertension additionally adjusted for sex, baseline smoking, BMI, and diabetes. HR for diabetes additionally adjusted for sex, baseline smoking, BMI, SBP, and DBP. Events are reported for the exposed vs. the unexposed group. Baseline smoking and updated smoking excludes occasional and ex‐regular smokers. For baseline smoking, current smokers were defined as regular smokers at baseline. For updated smoking, current smokers were defined as regular smokers or ex‐regular smokers who quit smoking ≤5 years ago at baseline.

There was no evidence for associations of obesity, hypertension, or diabetes with PD (*P* all > 0.05; Fig. 3). There was modest evidence for an association of current smoking with PD following adjustment for age, sex, and region. Men categorized as current vs. men categorized as never smokers had 39% lower risk (HR: 0.61: 0.44–0.86; Table S5). However, there were no associations of smoking with PD following adjustment for other confounders (*P *>* *0.05; Fig. 3).

Sensitivity analyses excluding the first 3 years of follow‐up did not alter the observed associations of smoking, obesity, hypertension, and diabetes with PD. However, sensitivity analysis revealed a higher risk of PD for overweight or obese vs. nonoverweight or obese participants (HR: 1.27: 95% CI: 1.04–1.56; Table S6). Analyses of overweight or obesity with PD in the subset of never smokers after adjustment for confounders and by exclusion of the first 3 years of follow‐up were unaltered. Assuming overweight or obesity status is causal for an increased risk of PD, population attributable fractions based on the fully adjusted HR indicate that overweight or obesity accounted for approximately 7.9% of all hospitalized cases of PD.

## Discussion

The results of this large study indicated that the age‐ and sex‐specific incidence rates of PD in Chinese population studies are broadly similar to those reported in Western populations.^9^, ^36^, ^40^ Consistent with findings in Western and Asian populations,^41^, ^42^, ^43^ a prior history of CVD in the CKB study was associated with a higher risk of PD, but there were no such associations with diabetes and hypertension.^14^, ^16^, ^17^ Consistent with results from previous reports in other populations,^2^, ^9^, ^11^, ^19^, ^21^, ^22^, ^23^, ^24^, ^27^, ^28^, ^29^, ^30^, ^44^ tobacco smoking was inversely associated with risk of PD, but this association was attenuated and no longer significant after adjustment for other confounders. Furthermore, risk of PD was higher among ex‐regular smokers who quit due to illness when compared with either current or never smokers, but there was no effect among other ex‐regular smokers.

While the results of previous prospective studies of BMI and risk of PD have been conflicting,^14^, ^18^, ^20^, ^26^, ^27^, ^29^ this study demonstrated a significant association of higher BMI with risk of PD. Assuming causality, a prior medical history of stroke and overweight or obesity accounted for approximately one‐sixth of PD risk in the CKB study. Hence, a large proportion of PD risk is unexplained by established risk factors for CVD, and further studies are required to assess the relevance of other risk factors for PD.

While results of previous case‐control studies were constrained by reverse causality bias, few prospective studies have assessed associations of CVD and CVD risk factors with PD, especially in Asian populations. Hence, the results from this study are informative and should guide public health recommendations to lower the incidence of PD in China by reducing the risk of stroke and levels of obesity.

However, this study had several limitations. While the CKB study involved analysis of 0.5 million individuals, there were only 521 PD cases in the subset without a prior history of CVD, and analyses of smoking were restricted to only 247 PD cases in men. Previous cohort studies, systematic reviews, and meta‐analyses of population studies in both Western and Asian populations reported that incidence rates of PD were higher in rural than in urban regions.^9^, ^35^, ^36^, ^45^ Ascertainment of PD cases in the CKB study included both hospitalizations and death from PD. However, none of the PD cases were validated, and it is possible that there may have been some under‐reporting of incident PD cases who were not admitted to hospital. Moreover, it was not possible to fully exclude some misclassification of PD from other cases with Parkinsonism, particularly in community hospitals, nor was it possible to exclude ascertainment bias in the diagnosis of PD in urban than in rural hospitals.

PD is a disease of late onset,^9^, ^35^ therefore associations may not have been observed with certain CVD risk factors due to the relatively short duration of follow‐up (10 years). This is especially relevant for analyses of associations of cigarette smoking with PD. China currently consumes over one‐third of the world's cigarettes, but the current high prevalence of smoking may have been too recent to assess the full effects of lifelong continued smoking at a population level.^4^, ^46^, ^47^, ^48^ Large‐scale genome‐wide association studies of PD cases are also needed for Mendelian randomization analyses to assess the causal relevance of the observed associations with modifiable risk factors.^49^, ^50^ However, this study suggests that reducing the levels of adiposity and other risk factors for stroke should also reduce the risk of PD.

## Author Contributions

JK designed this study, conducted the data analysis, drafted the manuscript, and conducted a critical review of the completed manuscript. SL and RC also designed the study and, together with BM‐K provided advice on data analysis and critical review of the completed manuscript. IT, YC, and LY conducted outcome adjudication and provided critical review of the completed manuscript. YG and ZB supervised data collection and provided critical review of the completed manuscript. ZC is the Principal Investigator of the CKB study, supervised data collection, and provided critical review of the completed manuscript.

## Conflicts of Interest

None of the authors report any conflicts of interest or have any financial disclosures.

## Supporting information


**Table S1.** Selected characteristics of China Kadoorie Biobank participants
**Table S2.** Associations of smoking with Parkinson's disease^a^
**Table S3**. Associations of smoking with incidence of Parkinson's disease after adjustment to follow‐up time^a^
**Table S4**. Associations of body mass index and weight with incidence of Parkinson's disease
**Table S5.** Associations of smoking, obesity, hypertension, and diabetes with incidence of Parkinson's disease
**Table S6.** Associations of smoking and adiposity with incidence of Parkinson's disease

## Data Availability

Anonymized individual participant data on baseline characteristics, first resurvey, and cause‐specific mortality from the CKB study participants are shared on request with *bona fide* medical researchers. Requests for additional data are limited to collaborative projects with CKB investigators. Details of study protocol and data access policies and procedures are available at http://www.ckbiobank.org.
