# Supplementary material for: Cardiovascular risk factors and Parkinson's disease in 500,000 Chinese adults
Source: Ann Clin Transl Neurol. 2019 Mar 10;6(4):624–32. doi: 10.1002/acn3.732 (PMC6469341; doi:10.1002/acn3.732)
Supplement: Supplementary file 1 — Table S1. Selected characteristics of China Kadoorie Biobank participants Table S2. Associations of smoking with Parkinson's diseasea Table S3. Associations of smoking with incidence of Parkinson's disease after adjustment to follow‐up timea Table S4. Associations of body mass index and weight with incidence of Parkinson's disease Table S5. Associations of smoking, obesity, hypertension, and diabetes with incidence of Parkinson's disease Table S6. Associations of smoking and adiposity with incidence of Parkinson's disease [file ACN3-6-624-s001.docx]

**Supplementary Material**

**Cardiovascular risk factors and Parkinson’s disease in 500,000 Chinese adults**

**Table of Contents**

|  |  | Page |
| --- | --- | --- |
| Table S1 | Selected characteristics of China Kadoorie Biobank participants | 2 |
| Table S2 | Associations of smoking with Parkinson’s disease | 3 |
| Table S3 | Associations of smoking with incidence of Parkinson’s disease after adjustment to follow-up time | 4 |
| Table S4 | Associations of body mass index and weight with incidence of Parkinson’s disease | 5 |
| Table S5 | Associations of smoking, obesity, hypertension, and diabetes with incidence of Parkinson’s disease | 6 |
| Table S6 | Associations of smoking and adiposity with incidence of Parkinson’s disease | 7 |

| **Table S1.** Selected characteristics of China Kadoorie Biobank participants | | | |
| --- | --- | --- | --- |
|  |  | **No prior cardiovascular disease** | |
| **Mean ± SD or No. (%)** | **All participants** | **All participants** | **Incident cases of Parkinson’s disease** |
| **Number of participants** | 503,497 | 480,950 | 521 |
| **Demographics** |  |  |  |
| **Age (years)** | 51.5 ± 10.7 | 51.0 ± 10.5 | 60.9 ± 8.9 |
| **Women** | 298,267 (59.2) | 285,506 (59.4) | 274 (52.6) |
| **Rural** | 281,991 (56.0) | 273,791 (56.9) | 287 (55.1) |
| **Income ≤ 19,999 Yuan** | 288,272 (57.3) | 275,046 (57.2) | 279 (53.6) |
| **Education ≤ primary school** | 255,819 (50.8) | 244,611 (50.9) | 324 (62.2) |
| **Agricultural worker or retired** | 293,411 (58.3) | 277,790 (57.8) | 357 (68.5) |
| **Lifestyle** |  |  |  |
| **Smoking (men)** |  |  |  |
| **Never** | 29,706 (14.5) | 27,857 (14.2) | 54 (21.8) |
| **Occasional** | 23,128 (11.2) | 22,240 (11.4) | 32 (13.0) |
| **Ex-regular** | 26,842 (13.1) | 24,008 (12.3) | 58 (23.5) |
| **Current** | 125,554 (61.2) | 121,339 (62.1) | 103 (41.7) |
| **Cigarettes per day^a^** |  |  |  |
| **0** | 27,857 (18.7) | 27,857 (18.6) | 54 (34.4) |
| **<20** | 54,405 (36.5) | 54,405 (36.5) | 53 (33.8) |
| **20+** | 66,934 (44.8) | 66,934 (44.9) | 50 (31.8) |
| **Alcohol consumption** |  |  |  |
| **Never regular** | 231,414 (46.0) | 220,388 (45.8) | 272 (52.2) |
| **Ex-regular** | 8,871 (1.8) | 7,586 (1.6) | 23 (4.4) |
| **Occasional/seasonal** | 160,570 (31.9) | 154,092 (32.0) | 138 (26.5) |
| **Monthly** | 17,031 (3.4) | 16,583 (3.5) | 10 (1.9) |
| **Reduced intake** | 11,324 (2.2) | 10,255 (2.1) | 15 (2.9) |
| **Weekly** | 74,287 (14.7) | 72,046 (15.0) | 63 (12.1) |
| **Physical activity (MET-hours/day)** | **21.1 ± 13.9** | **21.6 ± 13.9** | **14.8 ± 11.8** |
| **Medical History** |  |  |  |
| **Diabetes^b^** | 29,601 (5.9) | 25,813 (5.4) | 40 (7.7) |
| **Hypertension^c^** | 168,750 (33.5) | 154,614 (32.2) | 239 (45.9) |
| **Ischemic heart disease** | 15,082 (3.0) | - | - |
| **Stroke/transient ischemic attack** | 8,650 (1.7) | - | - |
| **Clinical Examination** |  |  |  |
| **Weight (kg)** | 59.8 ± 10.7 | 59.6 ± 10.7 | 59.5 ± 10.7 |
| **Height (cm)** | 158.7 ± 8.3 | 158.7 ± 8.3 | 157.9 ± 8.2 |
| **Body mass index (kg/m^2^)** | 23.7 ± 3.4 | 23.6 ± 3.3 | 23.8 ± 3.4 |
| **Waist-hip ratio** | 0.88 ± 0.07 | 0.88 ± 0.07 | 0.89 ± 0.07 |
| **Systolic blood pressure (mmHg)** | 131.1 ± 21.2 | 130.6 ± 21.0 | 135.5 ± 21.0 |
| **Diastolic blood pressure (mmHg)** | 77.8 ± 11.1 | 77.7 ± 11.1 | 77.8 ± 10.6 |
| **Follow-up (years)** | **9.0 ± 1.6** | **9.0 ± 1.6** | **6.0 ± 2.5** |
| ^a^Excludes ex-smokers and occasional smokers. ^b^Medical history or blood glucose detection of diabetes at baseline. ^c^SBP ≥140mmHg, DBP ≥90mmHg, or receiving treatment for hypertension. | | | |

| **Table S2.** Associations of smoking with Parkinson’s disease^a^ | | | | | |
| --- | --- | --- | --- | --- | --- |
|  | |  | **HR (95% CI**^b^**)** | | |
|  | **Events/**  **participants** | | **Adjusted for age-at-risk, region** | **+Income, education, occupation** | **Fully**  **adjusted**^c^ |
| **Baseline smoking** |  | |  |  |  |
| **Never** | 54/27,857 | | 1.00 (0.76-1.31) | 1.00 (0.76-1.31) | 1.00 (0.76-1.32) |
| **Occasional** | 32/22,240 | | 1.01 (0.71-1.42) | 1.09 (0.77-1.53) | 1.16 (0.82-1.64) |
| **Ex-regular** | 58/24,008 | | 1.05 (0.81-1.36) | 1.13 (0.87-1.46) | 1.13 (0.86-1.47) |
| **Current^d^** | 103/121,339 | | 0.61 (0.50-0.75) | 0.72 (0.58-0.88) | 0.77 (0.63-0.96) |
| ***Heterogeneity*^e^ *(***$\boldsymbol{\chi}^{\boldsymbol{2}}$***, P)*** | - | | 14.84, 0.002 | 9.21, 0.027 | 6.62, 0.085 |
|  |  | |  |  |  |
| **Updated smoking** |  | |  |  |  |
| **Never** | 54/27,857 | | 1.00 (0.76-1.30) | 1.00 (0.76-1.31) | 1.00 (0.76-1.32) |
| **Occasional** | 32/22,240 | | 1.00 (0.71-1.42) | 1.08 (0.77-1.53) | 1.16 (0.82-1.64) |
| **Quit due to illness** | 28/6,313 | | 1.68 (1.16-2.44) | 1.84 (1.27-2.67) | 1.83 (1.25-2.66) |
| **Other ex-regular** | 18/8,138 | | 0.93 (0.59-1.49) | 0.95 (0.59-1.51) | 0.98 (0.61-1.56) |
| **Current^d^** | 115/130,896 | | 0.61 (0.50-0.74) | 0.71 (0.58-0.87) | 0.76 (0.62-0.93) |
| ***Heterogeneity*^e^ *(***$\boldsymbol{\chi}^{\boldsymbol{2}}$***, P)*** | - | | 24.38, <0.001 | 18.69, <0.001 | 16.04, 0.003 |
|  |  | |  |  |  |
| **Cigarettes per day^f^** |  | |  |  |  |
| **0** | 54/27,857 | | 1.00 (0.76-1.31) | 1.00 (0.76-1.32) | 1.00 (0.75-1.33) |
| **1-19** | 53/54,405 | | 0.56 (0.36-0.86) | 0.73 (0.56-0.96) | 0.80 (0.61-1.05) |
| **20+** | 50/66,934 | | 0.61 (0.46-0.81) | 0.70 (0.52-0.94) | 0.75 (0.56-1.00) |
| ***Trend*^g^ *(P)*** | - | | 0.015 | 0.086 | 0.168 |

^a^Reported values are for men only. ^b^Hazard ratios (HR) are reported with group-specific 95% CIs. ^c^HR adjusted for age-at-risk, region, education, occupation, income, alcohol consumption, physical activity, BMI, SBP, DBP, and diabetes. ^d^For baseline smoking, current smokers were defined as regular smokers at baseline. For updated smoking, current smokers were defined as regular smokers or ex-regular smokers who quit smoking ≤5 years ago at baseline. ^e^Test for heterogeneity of HR by baseline and updated smoking categories, conducted using likelihood ratio tests with 3 and 4 degrees of freedom, respectively. ^f^Excludes ex-smokers and occasional smokers. ^g^Test for log linear trend of PD incidence HR across cigarette per day categories, conducted using a likelihood ratio test with 1 degree of freedom.

| Table S3. Associations of smoking with incidence of Parkinson’s disease after adjustment to follow-up time^a^ | | | | | | |
| --- | --- | --- | --- | --- | --- | --- |
|  |  | |  | | HR (95% CI^b^) | |
|  | | Standard events/  participants | | Updated  events/  participants | Fully  adjusted^c^ | +Removal of  first three years of follow-up^c^ |
| Baseline smoking | |  | |  |  |  |
| Never | | 54/27,857 | | 47/27,308 | 1.00 (0.76-1.32) | 1.00 (0.74-1.35) |
| Occasional | | 32/22,240 | | 30/21,868 | 1.16 (0.82-1.64) | 1.28 (0.89-1.83) |
| Ex-regular | | 58/24,008 | | 50/23,191 | 1.13 (0.86-1.47) | 1.12 (0.84-1.48) |
| Current^d^ | | 103/121,339 | | 86/118,692 | 0.77 (0.63-0.96) | 0.80 (0.64-1.01) |
| *Heterogeneity*^e^ *(*$\boldsymbol{\chi}^{\boldsymbol{2}}$*, P)* | | - | | - | 6.62, 0.085 | 5.81, 0.121 |
|  | |  | |  |  |  |
| Updated Smoking | |  | |  |  |  |
| Never | | 54/27,857 | | 47/27,308 | 1.00 (0.76-1.32) | 1.00 (0.74-1.35) |
| Occasional | | 32/22,240 | | 30/21,868 | 1.16 (0.82-1.64) | 1.27 (0.89-1.82) |
| Quit due to illness | | 28/6,313 | | 22/6,068 | 1.83 (1.25-2.66) | 1.63 (1.07-2.48) |
| Other ex-regular | | 18/8,138 | | 97/127,848 | 0.98 (0.61-1.56) | 1.03 (0.64-1.67) |
| Current^d^ | | 115/130,896 | | 17/7,967 | 0.76 (0.62-0.93) | 0.79 (0.64-0.99) |
| *Heterogeneity*^e^ *(*$\boldsymbol{\chi}^{\boldsymbol{2}}$*, P)* | | - | | - | 16.04, 0.003 | 10.04, 0.031 |
|  | |  | |  |  |  |
| Cigarettes per day^f^ | |  | |  |  |  |
| 0 | | 54/27,857 | | 47/27,308 | 1.00 (0.75-1.33) | 1.00 (0.74-1.35) |
| 1-19 | | 53/54,405 | | 41/52,947 | 0.80 (0.61-1.05) | 0.75 (0.55-1.02) |
| 20+ | | 50/66,934 | | 45/65,745 | 0.75 (0.56-1.00) | 0.86 (0.63-1.17) |
| *Trend*^g^ *(P)* | | - | | - | 0.168 | 0.595 |
| Standard events are all events occurring throughout the duration of follow up. Updated events are all events occurring after exclusion of the first three years of follow up. ^a^Reported values are for men only. ^b^HR are reported with group-specific 95% CIs. ^c^HR adjusted for age-at-risk, region, education, occupation, income, alcohol consumption, physical activity, BMI, SBP, DBP, and diabetes. ^d^For baseline smoking, current smokers were defined as regular smokers at baseline. For updated smoking, current smokers were defined as regular smokers or ex-regular smokers who quit smoking ≤5 years ago at baseline. ^e^Test for heterogeneity of HR by baseline and updated smoking categories, conducted using likelihood ratio tests with 3 and 4 degrees of freedom, respectively. ^f^Excludes ex-smokers and occasional smokers. ^g^Test for log linear trend of PD incidence HR across cigarette per day categories, conducted using a likelihood ratio test with 1 degree of freedom. | | | | | | |

| Table S4. Associations of body mass index and weight with incidence of Parkinson’s disease | | | | | |
| --- | --- | --- | --- | --- | --- |
|  |  |  | | **HR (95% CI**^a^**)** | |
|  | **Standard events/**  **participants** | | **Updated events/**  **participants** | **Fully**  **adjusted**^b^ | **+Removal of**  **first three years of follow-up**^b^ |
| Weight (kg) |  | |  |  |  |
| <50.6 | 121/98,027 | | 99/94,108 | 1.00 (0.82-1.22) | 1.00 (0.80-1.25) |
| 50.6 – 55.9 | 84/95,139 | | 71/95,201 | 0.97 (0.78-1.19) | 0.91 (0.72-1.15) |
| 56.0 – 61.2 | 95/96,732 | | 79/95,442 | 1.08 (0.88-1.32) | 1.05 (0.85-1.31) |
| 61.3 – 68.0 | 121/96,0033 | | 102/93,729 | 1.40 (1.17-1.68) | 1.39 (1.14-1.69) |
| ≥68.1 | 100/95,019 | | 95/95,038 | 1.32 (1.07-1.61) | 1.37 (1.09-1.71) |
| *Trend^c^ (P)* | - | | - | 0.006 | 0.007 |
|  |  | |  |  |  |
| Weight (kg) (NS^d^) |  | |  |  |  |
| <50.6 | 91/75,173 | | 78/73,934 | 1.00 (0.79-1.26) | 1.00 (0.78-1.28) |
| 50.6 – 55.9 | 52/67,308 | | 42/66,711 | 0.82 (0.63-1.08) | 0.79 (0.58-1.07) |
| 56.0 – 61.2 | 56/62,355 | | 45/61,830 | 0.98 (0.76-1.28) | 0.94 (0.71-1.26) |
| 61.3 – 68.0 | 71/53,930 | | 62/53,448 | 1.42 (1.13-1.80) | 1.48 (1.15-1.91) |
| ≥68.1 | 44/40,680 | | 39/40,310 | 1.13 (0.83-1.55) | 1.20 (0.86-1.68) |
| *Trend^c^ (P)* | - | | - | 0.076 | 0.051 |
|  |  | |  |  |  |
| BMI (kg/m^2^) |  | |  |  |  |
| <20.9 | 105/107,234 | | 82/104,506 | 1.00 (0.82-1.23) | 1.00 (0.79-1.26) |
| 20.9 – 22.8 | 109/110,383 | | 91/108,760 | 1.19 (0.99-1.44) | 1.29 (1.05-1.58) |
| 22.9 – 24.3 | 91/83,041 | | 79/82,068 | 1.31 (1.07-1.61) | 1.47 (1.18-1.83) |
| 24.5 – 26.6 | 111/95,285 | | 102/94,190 | 1.37 (1.14-1.65) | 1.63 (1.34-1.98) |
| ≥ 26.7 | 105/85,007 | | 92/83,994 | 1.48 (1.21-1.81) | 1.69 (1.36-2.09) |
| *Trend^c^ (P)* | - | | - | 0.005 | <0.001 |
|  |  | |  |  |  |
| BMI (kg/m^2^) (NS^d^) |  | |  |  |  |
| <20.9 | 61/61,533 | | 50/60,564 | 1.00 (0.77-1.30) | 1.00 (0.75-1.34) |
| 20.9 – 22.8 | 59/68,000 | | 47/67,321 | 1.03 (0.79-1.33) | 1.01 (0.76-1.35) |
| 22.9 – 24.3 | 53/52,844 | | 46/52,412 | 1.18 (0.91-1.55) | 1.29 (0.96-1.71) |
| 24.5 – 26.6 | 68/60,581 | | 62/60,009 | 1.28 (1.01-1.62) | 1.46 (1.14-1.87) |
| ≥ 26.7 | 73/56,488 | | 61/55,927 | 1.43 (1.12-1.81) | 1.52 (1.17-1.97) |
| *Trend^c^ (P)* | - | | - | 0.025 | 0.008 |
| Standard events are all events occurring throughout the duration of follow up. Updated events are all events occurring after exclusion of the first three years of follow up. ^a^HR are reported with group-specific 95% CIs. ^b^HR adjusted for age-at-risk, sex, region, income, education, occupation, alcohol consumption, physical activity, SBP, DBP, and diabetes. HR for weight and BMI in all participants additionally adjusted for baseline smoking. ^c^Test for log linear trend of PD incidence HR across quintiles of the main exposure, conducted using a likelihood ratio test with 4 degrees of freedom. ^d^NS is never smokers at baseline. | | | | | |

| **Table S5.** Associations of smoking, obesity, hypertension, and diabetes with incidence of Parkinson’s disease | | | | |
| --- | --- | --- | --- | --- |
|  |  | **HR (95% CI)** | | |
|  | **Events/**  **participants** | **Adjusted for age-at-risk, sex, region**^a^ | **+Income, occupation, education** | **Fully**  **adjusted**^b^ |
| **Baseline smoking (men)^c^** |  |  |  |  |
| **Never** | 54/27,857 | 1.00 (-) | 1.00 (-) | 1.00 (-) |
| **Current** | 103/121,339 | 0.61 (0.43-0.86) | 0.72 (0.50-1.03) | 0.78 (0.54-1.13) |
| ***Heterogeneity*^d^ *(***$\boldsymbol{\chi}^{\boldsymbol{2}}$***, P)*** | - | 7.47, 0.006 | 3.11, 0.078 | 1.68, 0.195 |
|  |  |  |  |  |
| **Updated smoking (men)^c^** |  |  |  |  |
| **Never** | 54/27,857 | 1.00 (-) | 1.00 (-) | 1.00 (-) |
| **Current** | 115/130,896 | 0.61 (0.44-0.86) | 0.72 (0.51-1.01) | 0.76 (0.53-1.09) |
| ***Heterogeneity*^d^ *(***$\boldsymbol{\chi}^{\boldsymbol{2}}$***, P)*** | - | 7.77, 0.005 | 3.46, 0.063 | 2.14, 0.144 |
|  |  |  |  |  |
| **Obesity** |  |  |  |  |
| **No** | 498/462,299 | 1.00 (-) | 1.00 (-) | 1.00 (-) |
| **Yes** | 23/18,651 | 1.16 (0.76-1.77) | 1.14 (0.75-1.74) | 1.18 (0.77-1.80) |
| ***Heterogeneity*^d^ *(***$\boldsymbol{\chi}^{\boldsymbol{2}}$***, P)*** | - | 0.47, 0.492 | 0.37, 0.544 | 0.54, 0.464 |
|  |  |  |  |  |
| **Hypertension** |  |  |  |  |
| **No** | 282/326,336 | 1.00 (-) | 1.00 (-) | 1.00 (-) |
| **Yes** | 239/154,614 | 0.99 (0.83-1.18) | 0.99 (0.83-1.18) | 0.93 (0.77-1.12) |
| ***Heterogeneity*^d^ *(***$\boldsymbol{\chi}^{\boldsymbol{2}}$***, P)*** | - | 0.01, 0.904 | 0.02, 0.897 | 0.62, 0.432 |
|  |  |  |  |  |
| **Diabetes** |  |  |  |  |
| **No** | 481/455,137 | 1.00 (-) | 1.00 (-) | 1.00 (-) |
| **Yes** | 40/25,813 | 1.00 (0.73-1.39) | 0.94 (0.68-1.30) | 0.93 (0.67-1.29) |
| ***Heterogeneity*^d^ *(***$\boldsymbol{\chi}^{\boldsymbol{2}}$***, P)*** | - | 0, 1.00 | 0.16, 0.687 | 0.20, 0.656 |
| ^a^All models except for baseline and updated smoking were adjusted for sex. ^b^HR further adjusted for alcohol consumption and physical activity. HR for baseline and updated smoking additionally adjusted for BMI, SBP, DBP, and diabetes. HR for obesity additionally adjusted for baseline smoking, SBP, DBP, and diabetes. HR for hypertension additionally adjusted for baseline smoking, BMI, and diabetes. HR for diabetes additionally adjusted for baseline smoking, BMI, SBP, and DBP. ^c^Excludes occasional and ex-regular smokers. For baseline smoking, current smokers were defined as regular smokers at baseline. For updated smoking, current smokers were defined as regular smokers or ex-regular smokers who quit smoking ≤5 years ago at baseline. ^d^Test for heterogeneity of HR by categories of the main exposure, conducted using a likelihood ratio test with 1 degree of freedom. | | | | |

| **Table S6.** Associations of smoking and adiposity with incidence of Parkinson’s disease | | | | |
| --- | --- | --- | --- | --- |
|  |  |  | **HR (95% CI)** | |
|  | **Standard events/**  **participants** | **Updated events/**  **participants** | **Fully**  **adjusted**^a^ | **+Removal of**  **first three years of follow-up**^a^ |
| **Baseline smoking (men)^b^** |  |  |  |  |
| **Never** | 54/27,857 | 47/27,308 | 1.00 (-) | 1.00 (-) |
| **Current** | 103/121,339 | 86/118,692 | 0.78 (0.54-1.13) | 0.83 (0.56-1.24) |
| ***Heterogeneity*^c^ *(***$\boldsymbol{\chi}^{\boldsymbol{2}}$***, P)*** | - | - | 1.68, 0.195 | 0.83, 0.362 |
|  |  |  |  |  |
| **Updated smoking (men)^b^** |  |  |  |  |
| **Never** | 57/27,857 | 47/27,308 | 1.00 (-) | 1.00 (-) |
| **Current** | 115/130,896 | 97/127,848 | 0.76 (0.53-1.09) | 0.81 (0.55-1.18) |
| ***Heterogeneity*^c^ *(***$\boldsymbol{\chi}^{\boldsymbol{2}}$***, P)*** | - | - | 2.14, 0.144 | 1.18, 0.277 |
|  |  |  |  |  |
| **Overweight/obesity** |  |  |  |  |
| **No** | 338/325,950 | 281/320,308 | 1.00 (-) | 1.00 (-) |
| **Yes** | 183/155,000 | 165/153,210 | 1.17 (0.98-1.41) | 1.27 (1.04-1.56) |
| ***Heterogeneity*^c^ *(***$\boldsymbol{\chi}^{\boldsymbol{2}}$***, P)*** | - | - | 2.58, 0.108 | 5.24, 0.022 |
|  |  |  |  |  |
| **Overweight/obesity (NS^d^)** |  |  |  |  |
| **No** | 191/198,516 | 159/196,272 | 1.00 (-) | 1.00 (-) |
| **Yes** | 123/100,930 | 107/99,961 | 1.27 (1.00-1.61) | 1.34 (1.04-1.73) |
| ***Heterogeneity*^c^ *(***$\boldsymbol{\chi}^{\boldsymbol{2}}$***, P)*** | - | - | 3.84, 0.050 | 4.91, 0.028 |
|  |  |  |  |  |
| **Obesity** |  |  |  |  |
| **No** | 498/462,299 | 426/455,099 | 1.00 (-) | 1.00 (-) |
| **Yes** | 23/18,651 | 20/18,419 | 1.18 (0.77-1.80) | 1.19 (0.75-1.87) |
| ***Heterogeneity*^c^ *(***$\boldsymbol{\chi}^{\boldsymbol{2}}$***, P)*** | - | - | 0.54, 0.464 | 0.52, 0.470 |
|  |  |  |  |  |
| **Obesity (NS^d^)** |  |  |  |  |
| **No** | 295/286,071 | 249/283,006 | 1.00 (-) | 1.00 (-) |
| **Yes** | 19/13,375 | 17/13,227 | 1.36 (0.85-2.17) | 1.45 (0.88-2.39) |
| ***Heterogeneity*^c^ *(***$\boldsymbol{\chi}^{\boldsymbol{2}}$***, P)*** | - | - | 1.48, 0.224 | 1.93, 0.165 |
| Standard events are all events occurring throughout the duration of follow up. Updated events are all events occurring after exclusion of the first three years of follow up. ^a^HR adjusted for age-at-risk, region, education, occupation, income, alcohol consumption, and physical activity. HR for baseline and updated smoking additionally adjusted for BMI, SBP, DBP, and diabetes. HR for overweight/obesity and obesity in all participants and in the subset of never smokers additionally adjusted for sex, SBP, DBP, and diabetes. HR for overweight/obesity and obesity in all participants additionally adjusted for baseline smoking. ^b^Excludes occasional and ex-regular smokers. For baseline smoking, current smokers were defined as regular smokers at baseline. For updated smoking, current smokers were defined as regular smokers or ex-regular smokers who quit smoking ≤5 years ago at baseline. ^c^Test for heterogeneity of HR by categories of the main exposure, conducted using a likelihood ratio test with 1 degree of freedom. ^d^NS is never smokers at baseline. | | | | |
